# Citrus Flavonoids as Antimicrobials

**DOI:** 10.1002/cbdv.202403210

**Published:** 2025-03-13

**Authors:** Rosaria Ciriminna, Giovanna Li Petri, Giuseppe Angellotti, Rafael Luque, Anne‐Sylvie Fabiano Tixier, Francesco Meneguzzo, Mario Pagliaro

**Affiliations:** ^1^ Istituto per lo Studio dei Materiali Nanostrutturati, CNR Palermo Italy; ^2^ Universidad Espíritu Santo (UEES) Samborondón Ecuador; ^3^ Avignon University, INRA, UMR 408, GREEN Team Extraction Avignon France; ^4^ Istituto per la Bioeconomia, CNR Sesto Fiorentino Italy

**Keywords:** antimicrobial resistance, citrus flavonoids, diosmin, hesperidin, naringenin, polymethoxyflavones

## Abstract

*Citrus* flavonoids are highly bioactive compounds exerting numerous health benefits including anticancer, antioxidant, antimicrobial, anti‐inflammatory, mitoprotective, and neuroprotective activity. Research on their broad‐scope bioactivity experienced a renaissance in the early 2000s, and further accelerated after COVID‐19, including research on their antimicrobial properties. Summarizing selected research achievements on the antimicrobial activity of the main *Citrus* flavonoids, this study aims to provide a unified picture on the antimicrobial properties of these valued compounds that will hopefully assist in the development of flavonoid‐based antimicrobials, including antibacterial treatments suitable for clinical use minimizing antimicrobial resistance.

## Graphical abstract

Citrus flavonoids are powerful and versatile antimicrobials whose mechanism of action often involves disruption of the bacterial membrane, thereby minimizing antimicrobial resistance. This study provides a unified perspective.

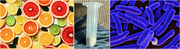

## 1 Introduction

Chiefly consisting of flavanones, flavones, flavonols (and anthocyanins, in blood oranges), *Citrus* flavonoids are highly bioactive compounds exerting anticancer, antioxidant, antimicrobial, anti‐inflammatory, mitoprotective, and neuroprotective activities, including positive effects on capillary fragility and ability to inhibit human platelet aggregation [1]. Today these biophenols comprise a “hot” field of research in biochemistry, agricultural and biological sciences, pharmacology, medicine and even in chemistry. A search with the query “citrus flavonoids” carried out on a research database shows that by the end of 2024, more than 14 000 (14 363) publications were indexed in the scholarly literature in English, the vast majority of which were articles (10 580) and reviews (2681) [2].

Current research interest spans from their nutritional value in healthy nutrition [3], to use as therapeutic agents for a number of ailments [4]. *Citrus* flavonoids, furthermore, exert antimicrobial activity for which they are used, for example, as active ingredients in commercial antimicrobials inhibiting the growth of a range of bacteria (and *Candida albicans* and *Candida dubliniensis* fungi) launched in the first decade of the 2000s [5]. Dubbed *Citrox*, the latter is a soluble formulation that contains nine bioflavonoids extracted from *Citrus aurantium* combined with organic acids, possessing strong antimicrobial, anti‐inflammatory, and antioxidative properties [6].

From bergamot [7] (*Citrus bergamia*), through grapefruit [8] (*Citrus paradisi*), lemon [8] (*Citrus limon*), and tangerine [8] (*Citrus reticulata*) numerous other *Citrus* fruits contain antimicrobial flavonoids. Binding to the phospholipids of bacterial membrane of multidrug resistant Gram‐positive bacteria, other plant flavonoids were recently shown to kill multidrug resistant bacteria [9]. Contrary to conventional antibiotics, whose mechanism of action is based on bacterial enzyme inhibition, their specific mode of action based on membrane disruption (with leakage of cell contents and breakdown of the transmembrane potential) prevents antimicrobial resistance (AMR) as the interaction of flavonoids with bacterial membrane is not mediated through a specific target [10]. Furthermore, certain *Citrus* flavonoids such as naringenin interfere with bacterial quorum sensing (QS) inhibiting pathogenic bacteria such as *Pseudomonas aeruginosa* [11]. Interfering with QS is another alternative route capable to minimize AMR [12]. Of particular urgency is the development of new antibacterial agents to combat bacterial AMR in ESKAPE (*Enterococcus faecium*, *Staphylococcus aureus*, *Klebsiella pneumoniae*, *Acinetobacter baumannii*, *P. aeruginosa*, and *Enterobacter* species) pathogenic bacteria involved in high mortality risk infections due to the acquisition of AMR genes by ESKAPE pathogens [13].

Summarizing research achievements on the antimicrobial activity of the main *Citrus* flavonoids, this study aims to provide a unified picture useful to develop flavonoid‐based antibacterial treatments, suitable for clinical use, minimizing AMR. After a brief background on *Citrus* flavonoids and scholarly research focusing on these compounds, in the following we focus on the antimicrobial activity of the main *Citrus* flavonoids, based on criteria of relative abundance in the most harvested *Citrus* fruits that are orange, tangerine, lemon and grapefruit. Flavonoids abundant in less common, but still widely cultivated fruits such as bergamot, are included for their relevant antimicrobial activity.

## 2 Citrus Flavonoids

**FIGURE 1:**
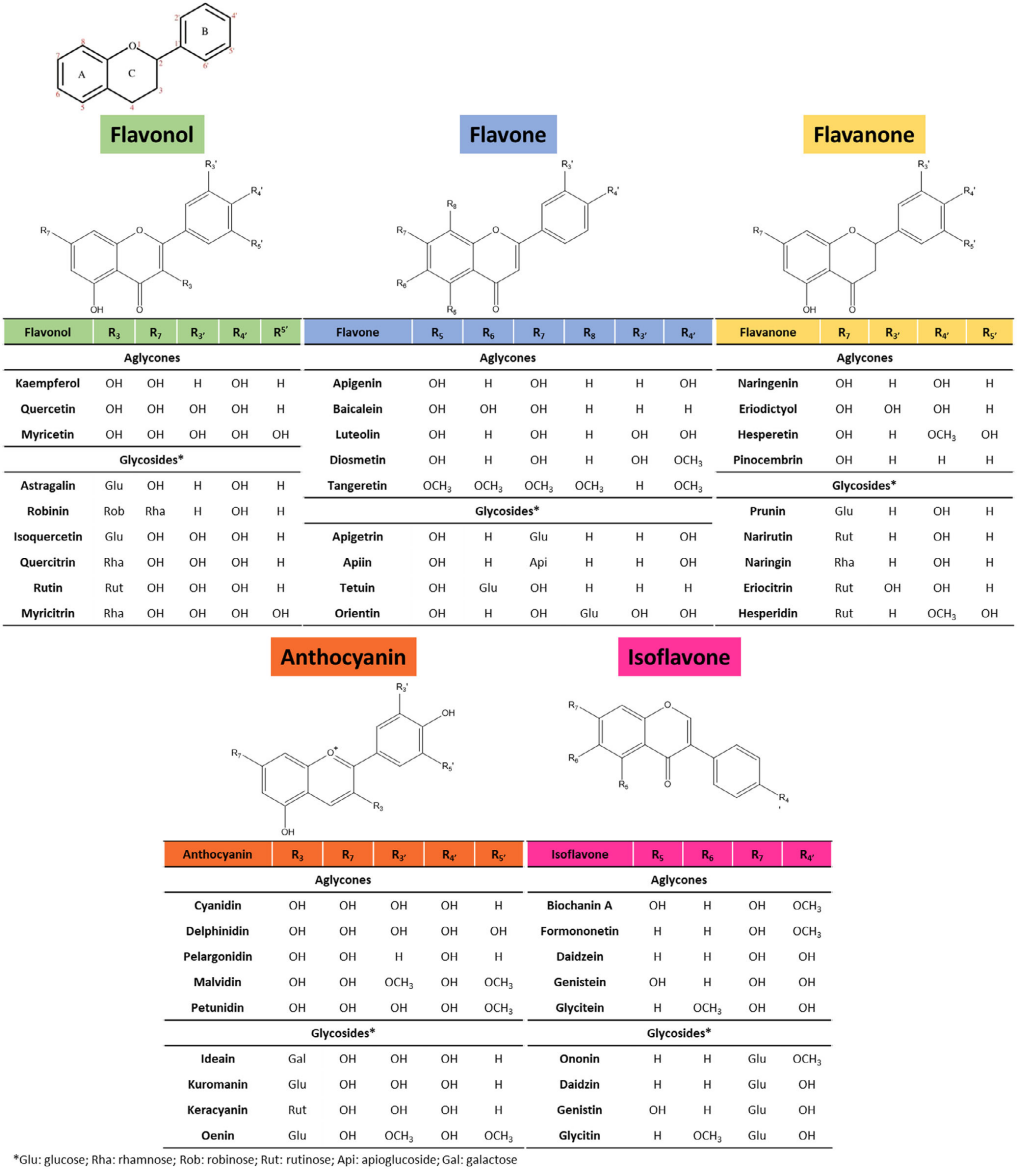
Flavonoid general structure (top). Flavonoid structures organized by their families. Adapted from [15], Creative Commons Attribution (CC BY) license.

Secondary metabolites used by plants as defense against microbes, insects, drought, UV radiation and other threats [14], flavonoids are polyphenolic molecules having a fundamental structure phenylpropanoid chain (C6–C3–C6), consisting of two aromatic rings (A and B) linked by a heterocyclic pyran ring (C). Except for isoflavones, these heterocyclic compounds have two benzene rings (A and B) bridged together by a heterocyclic pyrone (C) to which one aromatic ring is attached at C2. Flavones and flavonols have a double bond at C2 and C3, while flavonols are hydroxylated at C3 position. After oxidation they respectively become their respective dihydroxylated forms: flavanones and flavanonols [15]. Classification into flavonol, flavone, flavoanone, anthocyanin, and isoflavanone subgroups is based on cyclization and degree of oxidation in the C3 chain (Figure 1).

With the exception of anthocyanins present in blood oranges, *Citrus* flavonoids consist of flavanones, flavones, and flavonols. Examples of *Citrus* flavonols are quercetin and kaempferol. Examples of flavones are apigenin and diosmetin. Examples of flavanones (also called dihydroflavones) are hesperetin, naringenin, and eriodictyol.

**FIGURE 2:**
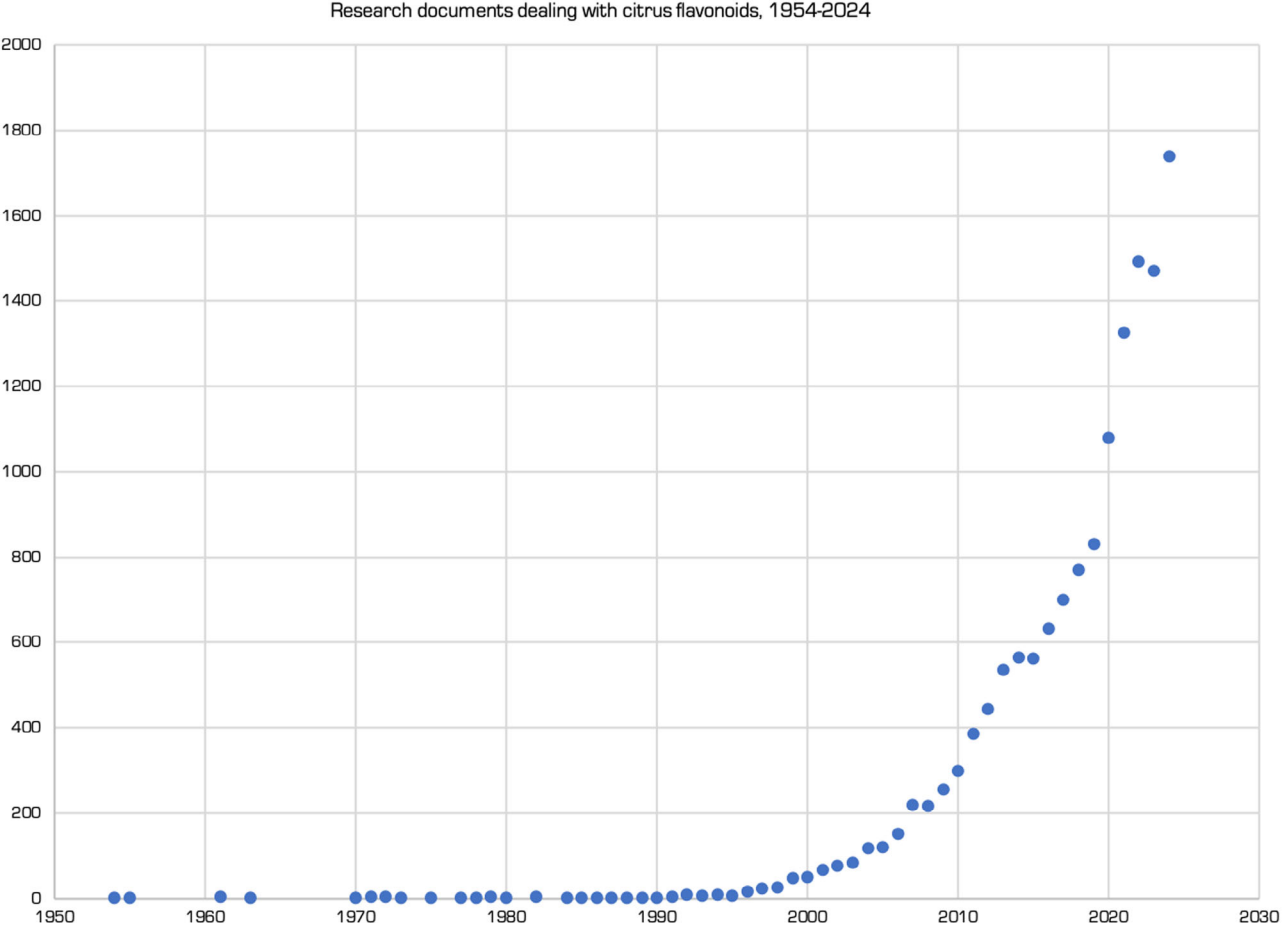
Documents by year dealing with “citrus flavonoids” indexed by research database Scopus between 1954 and late 2024. Created with data from scopus.com, with kind permission.

For nearly 50 years between 1954 and 1991 the interest of researchers for *Citrus* flavonoids remained negligibly low, with less than 3 (and often 0) articles/year published (Figure 2) [2]. After a first period of growth between 1996 and 2003, year 2004 was the first in which the number of publications exceeded the 100 threshold (117). However, it took only 3 more years to overcome the 200 threshold in 2007 (219 publications) and another 4 years to surpass the 300 publications threshold (386 in 2011). The COVID‐19 health crisis led to surpass the 1000 publications/year threshold in 2020 (1080 publications), followed by further substantial increase to 1326 publications in 2021 and to 1492 publications in 2022. Since then, citrus flavonoids remained a hot research topic also in 2023 (1470 publications) and in 2024 the number of annual publications reached the highest value ever (1739 publications) surpassing the peak observed in 2022 [2].

### 2.1 Naringin and Naringenin

**FIGURE 3:**
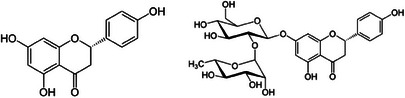
Chemical structures of naringenin (left) and naringin.

Found in particularly large amounts in the pulp and albedo (and to a lower extent also in the flavedo) of grapefruit (490–4100, 50–1050, and 940–2240 mg/100 g fresh weight) [16], and sour oranges in the form of its 7‐*O*‐glycoside naringin (4′,5,7‐trihydroxyflavanone‐7‐rhamnoglucoside), naringenin (Figure 3) (4′,5,7‐trihydroxyflavanone) has good antibacterial activity against Gram‐positive bacteria, and low activity against Gram‐negative bacteria, with MIC values ranging from 0.5 to 1 up to 2000 µg/mL [17]. In agreement with the fact that flavanones have modest antifungal activity, naringenin has low activity against both filamentous and sporulation fungi [17].

The first indirect proof that the antibacterial activity of poorly water soluble naringin is due to its destabilizing effects on the outer membrane of bacterial cells was obtained in 2011 by scholars in Argentina [18]. The team used enzymes to remove one rhamnose from naringin and to esterify the primary alcohol groups of the remaining rhamnose moiety in prunin. At low concentration of 0.25 mmol/L, the most hydrophobic alkyl prunin esters with an optimum number of carbon atoms in the fatty acid ester chain between 10 and 12 were the best antimicrobial agents especially against *Listeria monocytogenes* and *S. aureus*. Furthermore, antimicrobial activity of the prunin‐esters was significant against Gram‐positive bacteria virtually absent against Gram‐negative bacteria. The chain would provide more hydrophobicity to the glucosyl aglycone, which certainly would allow better interaction at the membrane level. A similar approach was followed by scholars in Poland incorporating *O*‐alkyl groups containing six‐carbon chains attached to the C7 and/or C4′ position, further modified incorporating an oxime group in place of the carbonyl moiety at the C4 position. The new compounds exhibited significantly increased activity against pathogenic microorganisms, both Gram‐positive (e.g., *S. aureus* and *E. faecalis*) and Gram‐negative (e.g., *Helicobacter pylori*, *Escherichia coli*, *P. aeruginosa*) [19].

Finally, a simpler and effective approach to enhanced antibacterial activity of naringin is achieved by loading the flavanone on suitable carrier materials to improve dissolution and thus facilitating diffusion of naringin into the bacterial cells. This has been shown using biocompatible and biodegradable polymer cyclodextrin [20] or amorphous hydrophilic polymers [21]. Investigation by atom force microscopy of *E. coli* bacteria contacted with β‐cyclodextrin nanoparticles (β‐CD NPs) clearly showed destructive changes in the bacterial cell's morphology through destabilization of outer membrane of bacterial cells. While no effect or slight damage of *E. coli* cells treated with empty β‐CD NPs or with pure naringin was observed, significant disruption of the bacterial cells was observed after treatment with microencapsulated naringin NR‐β‐CD. Similarly, treatment of *L. monocytogenes* with naringin affected smoothness of the cell surface only with full retention of the typical rod morphology, while complete destruction of cells occurred upon treatment with CD‐entrapped naringin. Finally, complete destruction of bunches as well as shape distortion in cells of *E. faecalis* cells was observed upon treatment with microencapsulated naringin.

Research on the antibacterial properties of naringin actively continues, especially with the aim to synergistically benefits from the numerous other health beneficial properties of the flavonoid. For example, scholars in China recently reported that naringin administered to mice having femurs infected with *S. aureus* (mouse model of osteomyelitis) alleviated *S. aureus*‐induced cortical bone destruction and bone loss in mice suppressing at the same time *S. aureus*‐induced bacterial growth and inflammation in femurs [22].

**FIGURE 4:**
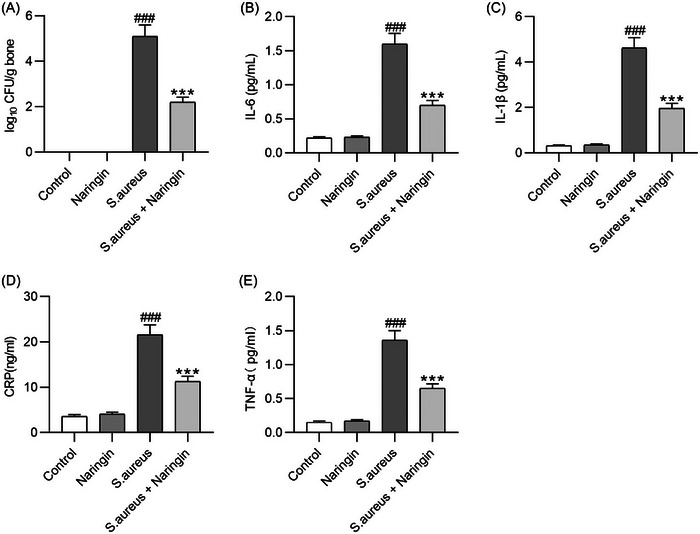
(A) Bacterial levels in the bones of mice from four experimental groups were determined. (B–E) Enzyme‐linked immunosorbent assay was performed to measure the levels of interleukin‐6 (B), IL‐1β (C), C‐reactive protein (D), and tumor necrosis factor‐α (E) in the femurs from each group. Reproduced from [22], with kind permission from John Wiley and Sons, 2024.

In detail, as shown in Figure 4A, the bones in the mice control group were not infected by bacteria, whereas *S. aureus* exposure led to a surge in bacterial levels in the bones of model mice. However, naringin treatment significantly suppressed bacterial growth. Stimulation by *S. aureus* resulted in the release of proinflammatory cytokines in mice, and IL‐6, IL‐1β, CRP, and TNF‐α levels were upregulated in mice in the *S. aureus* group versus those in the control group. Thus, naringin treatment significantly diminished the levels of these cytokines in the femurs of mice with osteomyelitis.

### 2.2 Tangeretin and Nobiletin

**FIGURE 5:**
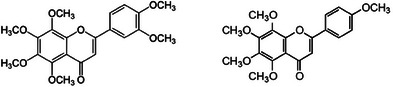
5 Chemical structures of nobiletin (left) and tangeretin.

Present also in the peel of orange [23] and other *Citrus* fruits, but particularly abundant in the peel of mandarin (*Citrus reticulate* Blanco) (up to 61 mg/100 g of dry mandarin peel weight for nobiletin and 25 mg/100 g of dry mandarin peel weight for tangeretin) [24], nobiletin (5,6,7,8,3′,4′‐hexamethoxyflavone; Figure 5) and tangeretin (5,6,7,8,4′‐pentamethoxyflavone) are the main citrus polymethoxyflavones (PMFs).

Studying the antibacterial mechanism of nobiletin and tangeretin against *Pseudomonas fluorescens* and *P. aeruginosa* in 2012 scholars in China found that the two PMFs destroy the permeability of the cell membrane, with release of the cell constituents, leading to metabolic dysfunction, inhibition of protein synthesis, and eventually to cell death [25].

**FIGURE 6:**
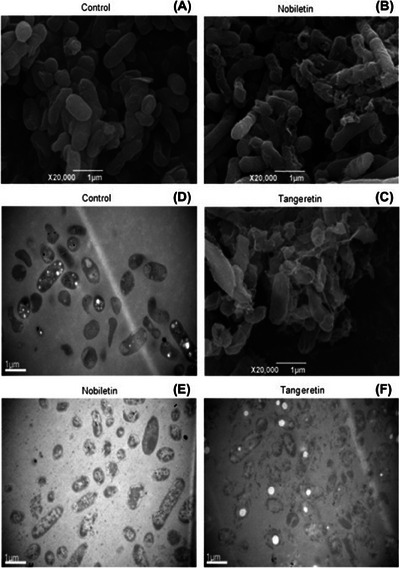
SEM (A–C) and TEM (D–F) photographs showing the effect of nobiletin and tangeretin on morphology of *Pseudomonas aeruginosa* cells. Reproduced from [25], with kind permission from Elsevier, 2012.

Electron microscopy showed that the structure of the bacterial cells was destroyed and accompanied with induced cells plasmolysis. Nobiletin and tangeretin furthermore inhibit the activities of succinate and malate dehydrogenases, and reduce proteins synthesis in bacterial cells. In detail, SEM and TEM observation of both control and PMFs treated bacterial cells show that bacterial cells treated with PMFs had damaged morphology with membranes disrupted in both bacteria. Serious plasmolysis was evident with depletion of *P. fluorescens* and *P. aeruginosa* cells contents, confirming that the cell membrane structures of both bacteria had been severely damaged by the PMFs. Bacterial cells treated with tangeretin exhibited more damage to cell membrane (Figure 6C,F), than those treated with nobiletin (Figure 6B,E). The TEM photographs clearly show that the membrane structure of both bacteria, namely that the primary target for both PMFs in *P. fluorescens* and *P. aeruginosa* is the cell membrane. In agreement, the team measured significant leakage of reducing sugar from intracellular to extracellular medium when the bacterial cells were treated with PMFs at a concentration of 3.6 mg/mL (MIC, v/v). Furthermore, both PMFs impaired the protein synthesis, with the treated cells of *P. aeruginosa* showing a significant reduction in intracellular protein concentrations from 2289.6 µg/mL for the control to 1397.2 and 1162.4 µg/mL in presence of nobiletin and tangeretin, respectively, leading to metabolic dysfunction and bacterial death.

The team concluded that their findings provided a new “approach to developing promising natural antimicrobial agents with potential applications in the food and pharmaceutical industries” [25]. Yet, surprisingly, little research on the antimicrobial activity of these two flavonoids was reported in the subsequent decade, with most research focusing instead on the anticancer properties of both PMFs [26].

Furthermore, these results showed evidence that lipophilic flavonoids such as citrus PMFs target the bacterial cell membrane of Gram‐negative bacterials, as it happens for plant flavonoids against Gram‐positive bacteria for which correlation exists between the antimicrobial activity physicochemical parameters log*P* (calculated partition coefficient) and logD7.40 (log_10_ of distribution coefficient at pH 7.40) [27].

**FIGURE 7:**
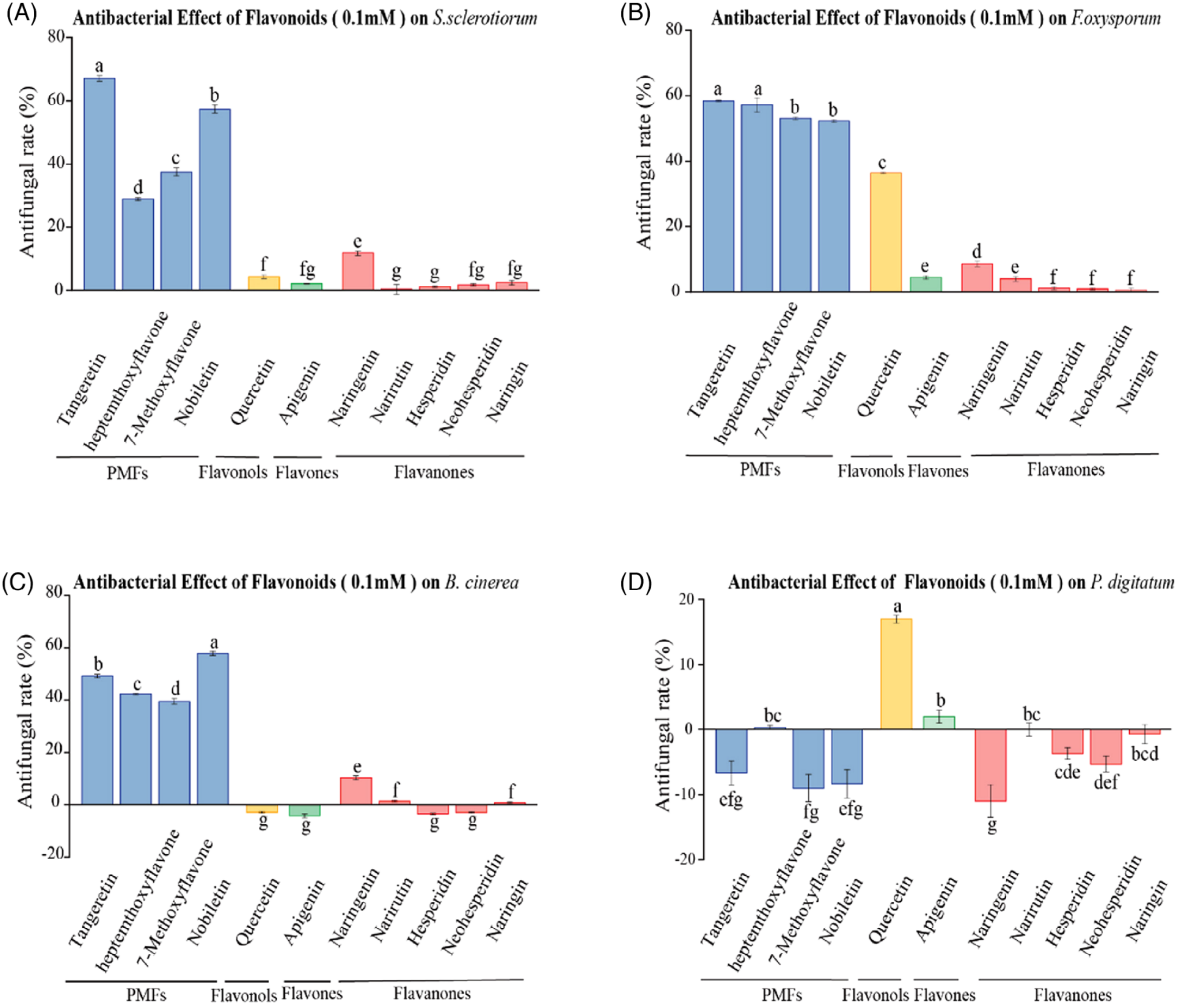
Antifungal activity of individual pure flavonoid compounds against four phytopathogens at 0.1 mM (A, *Sclerotinia sclerotiorum*; B, *Fusarium oxysporum* f. sp. *cucumerinum*; C, *Botrytis cinerea*; D, *Penicillium digitatum*). Reproduced from [27], Creative Commons Attribution 4.0 International License from The Authors, 2021.

A first major advance following the above findings dating back to 2012, was lately reported by another China‐based team describing the strong antifungal role of a hydroalcoholic extract of the peel of *C. reticulata* cultivar typical of in Guangdong, China (*C. reticulata* “Chachiensis”) [27]. This variety contains higher levels of PMFs, particularly nobiletin, compared to other citrus varieties [28]. Differences in antifungal activity among individual flavonoid compounds suggest that the inhibitory effect of citrus flavonoid extracts is primarily attributed to PMFs, while flavonoid glycosides contribute the least [27]. In detail, to assess the inhibitory activity of individual flavonoids, *Botrytis cinerea*, *Sclerotinia sclerotiorum*, *Fusarium oxysporum* f. sp. *cucumerinum*, and *Penicillium digitatum* were co‐cultivated with nine pure flavonoid compounds at concentration of 0.05 and 0.1 mM, respectively (Figure 7) [27].

Almost all of the flavonoid compounds exhibited varying degrees of inhibitory activity against these three non‐adapted fungi. Among these pure flavonoid compounds, the inhibitory activity of PMFs tangeretin and nobiletin against these three non‐adapted fungi was significantly higher than flavonols, flavones and flavanones at both concentrations. Narirutin, the glycosidic form of naringenin, showed a lower inhibitory effect, indicating that flavanones in their aglycone form demonstrate better activity. Consistent with the results of antifungal assays using citrus flavonoid extracts, none of these individual flavonoid compounds hindered the growth of the adapted fungus *P. digitatum* except quercetin (Figure 7D).

**FIGURE 8:**
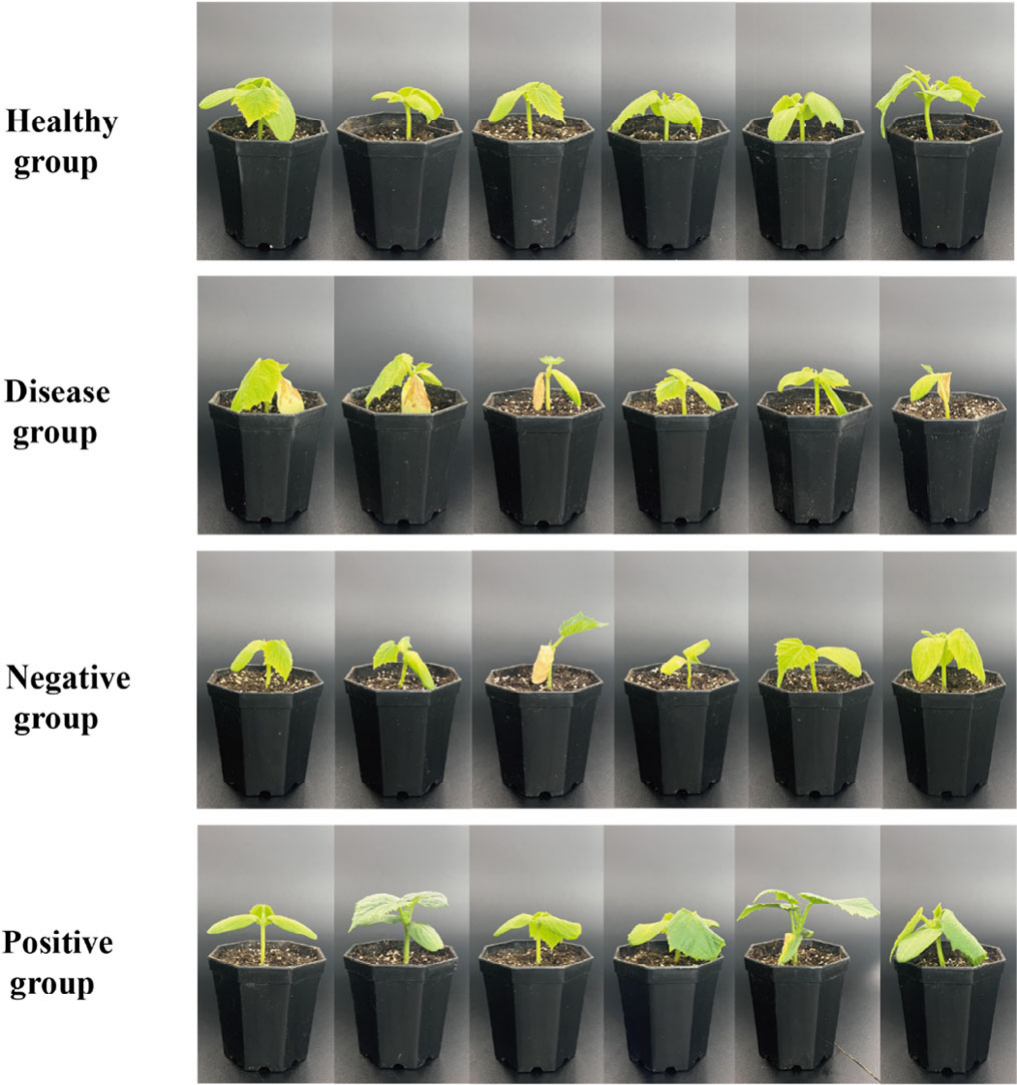
Photos of cucumber seedlings under four treatments after 20 days of cultivation in the greenhouse. Healthy group: cucumber seedlings without any treatment; disease group: cucumber seedlings inoculated with a fungal pathogen *Fusarium oxysporum* f. sp. *cucumerinum* and treated with sterilized water; negative group: cucumber seedlings inoculated with the pathogen and treated with empty microcapsules (microcapsules without flavonoid extracts); positive group: cucumber seedlings inoculated with the pathogen and treated with citrus flavonoid microcapsules. Reproduced from [27], Creative Commons Attribution 4.0 International License from The Authors, 2021.

The team went further and encapsulated the powdered hydroalcoholic extract of the peel of *C. reticulata* within microcapsules prepared using environmentally friendly wall materials sodium alginate and gelatin (optimal conditions: sodium alginate to gelatin ratio of 3:1, concentration of PMFs at 0.6 mg/mL, and 3% concentration of CaCl_2_ for cross‐linking). Photographs in Figure 8 showing five cucumber seedlings randomly selected from each group after 20 days of cultivation under various treatments provide evidence that plants treated with the microcapsules loaded with PMFs efficiently reducing disease incidence. The disease group had a disease severity index (DSI) of 57.16%, while the group treated with citrus flavonoid microcapsules had DSI 22.71%. Showing evidence of further beneficial effects of the microencapsulated PMFs as well as of the alginate/gelatin microcapsule wall materials, a slight growth‐promoting effect can be observed in cucumber seedlings treated with citrus flavonoid loaded and empty microcapsules.

### 2.3 Hesperidin and Hesperetin

**FIGURE 9:**
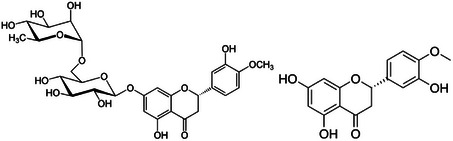
Chemical structures of hespiridin (left) and hesperetin.

A flavanone glycoside composed of the aglycone hesperetin (3,5,7‐trihydroxy 4′‐methoxyflavanone; Figure 9) and rutinose, hesperidin (hesperetin 7‐rutinoside) is abundant in the peel of several citrus fruits, particularly in the orange peel hesperidin is the major flavonoid glycoside found in the orange peel where its amount (48 mg/g of dry peel) makes it a commercial source of hesperidin [29]. In Argentina, the alcoholic extract (26% ethanol volume) made from bitter orange peel is a successful beverage commercialized as a flavored aperitif with health‐beneficial properties with tradename “Hesperidina” since 1864 [30].

Both hesperidin and hesperetin possess antimicrobial activity whose multifactorial mechanism involves bacterial membrane disruption, and inactivation of microbial enzymes [31]. In 2022, a joint industry‐academic team in South Korea reported important new results correlating lipophilicity with antimicrobial activity [32]. First, they confirmed that the aglycone hesperetin is substantially more active than hesperidin against both Gram‐positive and Gram‐negative bacteria tested. For example, hesperetin had minimal bactericidal concentration (MBC) = 500 µg/mL against *S. aureus*, whereas hesperidin had MBC > 2000 µg/mL. By derivatizing, hesperetin with glucose, however, the team reported that water soluble hesperidin glucoside showed antibacterial activities substantially higher (MBC = 1000 µg/mL against *S. aureus*) than poorly water‐soluble hesperidin. The team concluded that “vehiculation of the flavonoid moiety is the key factor explaining the antimicrobial activity of flavonoids” [32].

**FIGURE 10:**
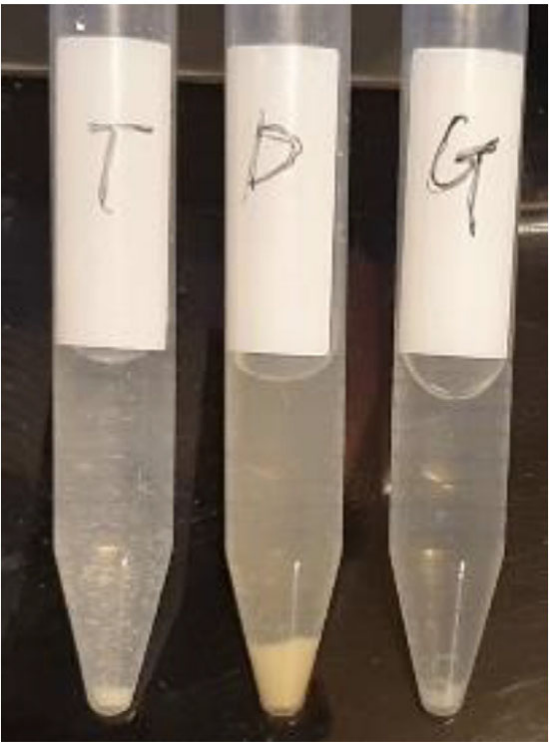
Microbial cells and in culture broth in the presence of hesperetin, hesperidin, and hesperidin glucoside. Reproduced from [32], Creative Commons Attribution (CC BY) license from The Authors, 2022.

In further detail, addition of 2% DMSO to the culture broth did not sufficiently increase the solubility of hesperidin or hesperetin, and there were some precipitates in the bacterial culture broth at high concentration of hesperidin or hesperetin. On the other hand, hesperidin glucoside was completely solubilized in the culture broth (Figure 10). The bactericidal activity of hesperidin glucoside was comparable to that of hesperetin when considering that the molecular weight of hesperidin glucoside (∼773 g/mol) is nearly twice than that of hesperetin (∼303 g/mol).

**FIGURE 11:**
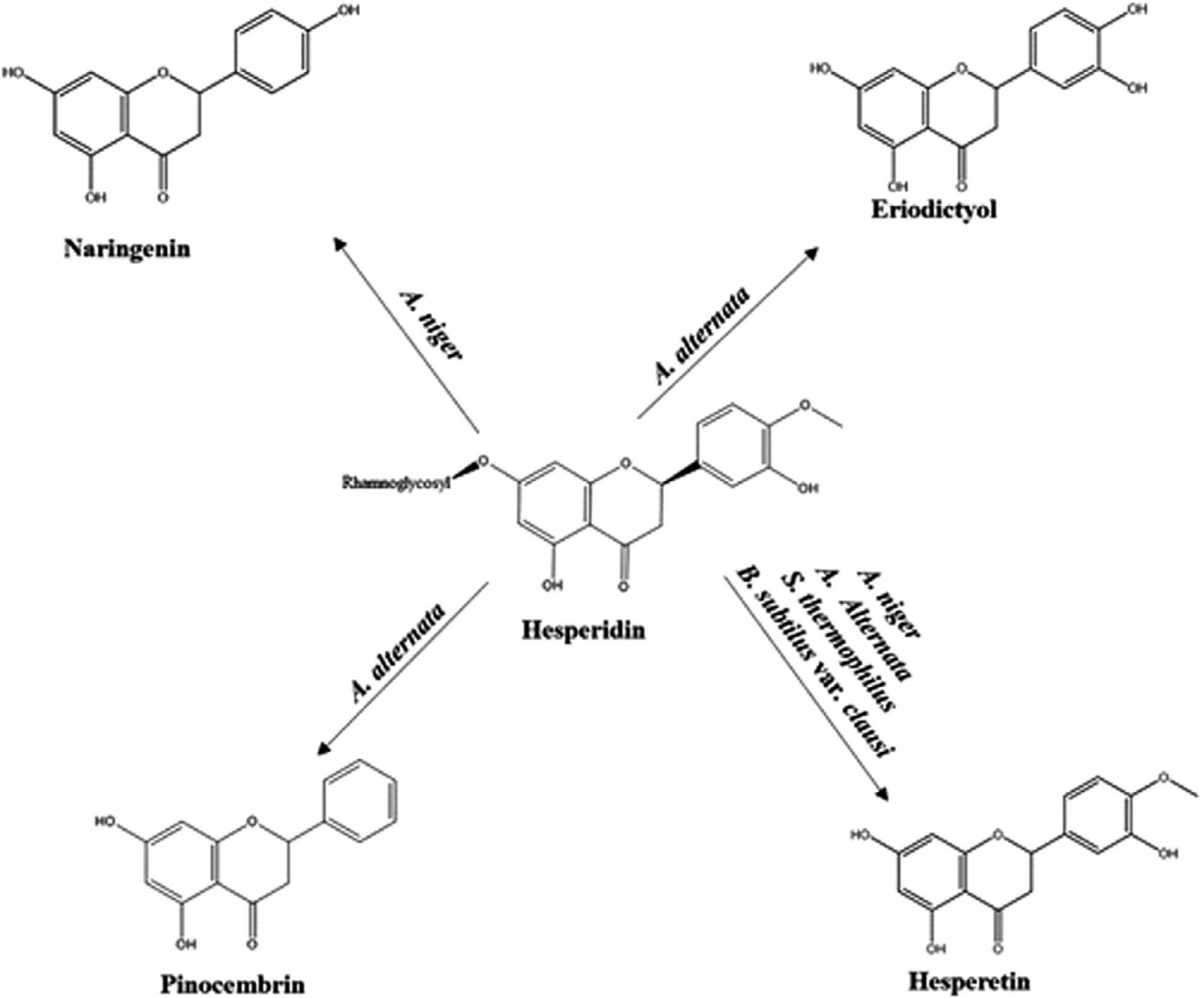
Microbial transformation of hesperidin established in 2023. Reproduced from [33], Creative Commons Attribution (CC BY) license from The Authors, 2023.

The subsequent year, another team based in Türkiye reported the first proof that hesperidin administered to 25 different nonhuman pathogenic microorganisms undergoes biotransformation into pinocembrin, naringenin, eriodictyol and hesperetin (Figure 11) [33].

Comparative antimicrobial tests of hesperidin and its metabolites confirmed that hesperetin is more active than hesperidin. Only the naringenin metabolite had a bactericidal effect against the *S. aureus* strain (at a concentration of 500 µg/mL). Hesperetin *and* naringenin, furthermore, showed antimicrobial activity against the human pathogenic *S. aureus* strain [33], with a significant synergistic effect on the biofilm plate.

### 2.4 Kaempferol

**FIGURE 12:**
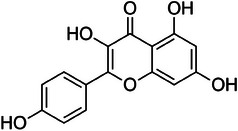
Chemical structure of kaempferol.

Kaempferol (3,5,7‐trihydroxy‐2‐(4‐hydroxyphenyl)‐4*H*‐1‐benzopyran‐4‐one; Figure 12) is a flavanol present in the lemon [34], orange and grapefruit peels as kaempferol‐3‐glucoside and in pure form in overall amounts between 5 µg/g (for lemon) and 10 µg/g (for orange and grapefruit) [35].

Owing its name to naturalist Engelbert Kaempfer (1651–1716), kaempferol has broad‐scope antimicrobial activity, [36] In contact with bacterial cells, kaempferol generates reactive oxygen species (ROS) through different mechanisms including interaction of its phenoxyl radical with oxygen [37].

Accordingly it is highly effective, for instance, in damaging the cell membrane of *E. coli*, causing bacterial protein leakage into the extracellular environment [38]. In detail, the normal cells of *E. coli* ATCC 25922 display the typical Gram‐negative structure with intact membrane and high density cytoplasm. Treatment of the same cells with kaempferol resulted in alteration of the morphology and integrity of cell membranes, with plasmolysis involving the outflow of intracellular constituents.

Using a model membrane used to mimic *E. coli* ATCC 25922, the team suggested antimicrobial mechanism of kaempferol should be ascribed to interaction with the hydrophilic region of phospholipids on cell membrane and eventual penetration of the hydrophobic core at increased flavonoids concentration. Another important antibacterial mechanism was demonstrated for *E. coli*, where kaempferol was shown to be the most effective flavonoid in directly inhibiting the bacterial DNA gyrase [39]. Kaempferol exhibited the greatest antibacterial activity (MIC = 25 µg/mL). The team went further and shed light on how the number and positions of substituted methoxyl and hydroxyl groups affect antibacterial activity of flavonoids by investigating the antibacterial mechanism of flavonoids against *E. coli* by inhibition assay of DNA gyrase. All 10 flavonoids tested showed inhibitory effects against *E. coli*.

However, flavonoids containing hydroxyl groups showed higher inhibitory values than the flavonoids with methoxyl groups. Kaempferol, which has hydroxyl group substitutions at C‐3, C‐5, C‐7, and C‐4, showed the highest activity (MIC = 25 µg/mL), while nobiletin, which has methoxyl group substitutions at C‐5, C‐6, C‐7, C‐8, C‐3′, and C‐4′, demonstrated the lowest activity (MIC = 177 µg/mL). The scholars found good correlation between the inhibitory effect of flavonoid on DNA gyrase assessed by determining the concentration of flavonoid required to inhibit 50% of the supercoiling activity of the enzyme (IC_50_), and the concentration that inhibits the growth of 50% of organisms (MIC_50_). Results showed that for good inhibitory effect the hydroxyl group at C‐5 in the A ring and C‐4′ in the B ring and the methoxyl group at C‐3 and C‐8 in the A ring are essential, while the presence of the hydroxyl group at C‐6 in the A ring, C‐3′ and C‐5′ in the B ring, and C‐3 in the C ring and the methoxyl group at C‐3′ in the B ring greatly reduced inhibition of bacteria. Similarly, kaempferol at low concentration (35 µM) binds to the PriA helicase in *S. aureus* and then inhibits its ATPase activity readily detected by measuring the concentration of inorganic phosphate released by ATP hydrolysis [40]. This is particularly relevant because PriA, an enzyme possessing ATPase and 3′–5′ helicase activities crucial for DNA replication restart, is not present in the human body, making the kaempferol targeting PriA inhibition intrinsically safe for human use.

**FIGURE 13:**
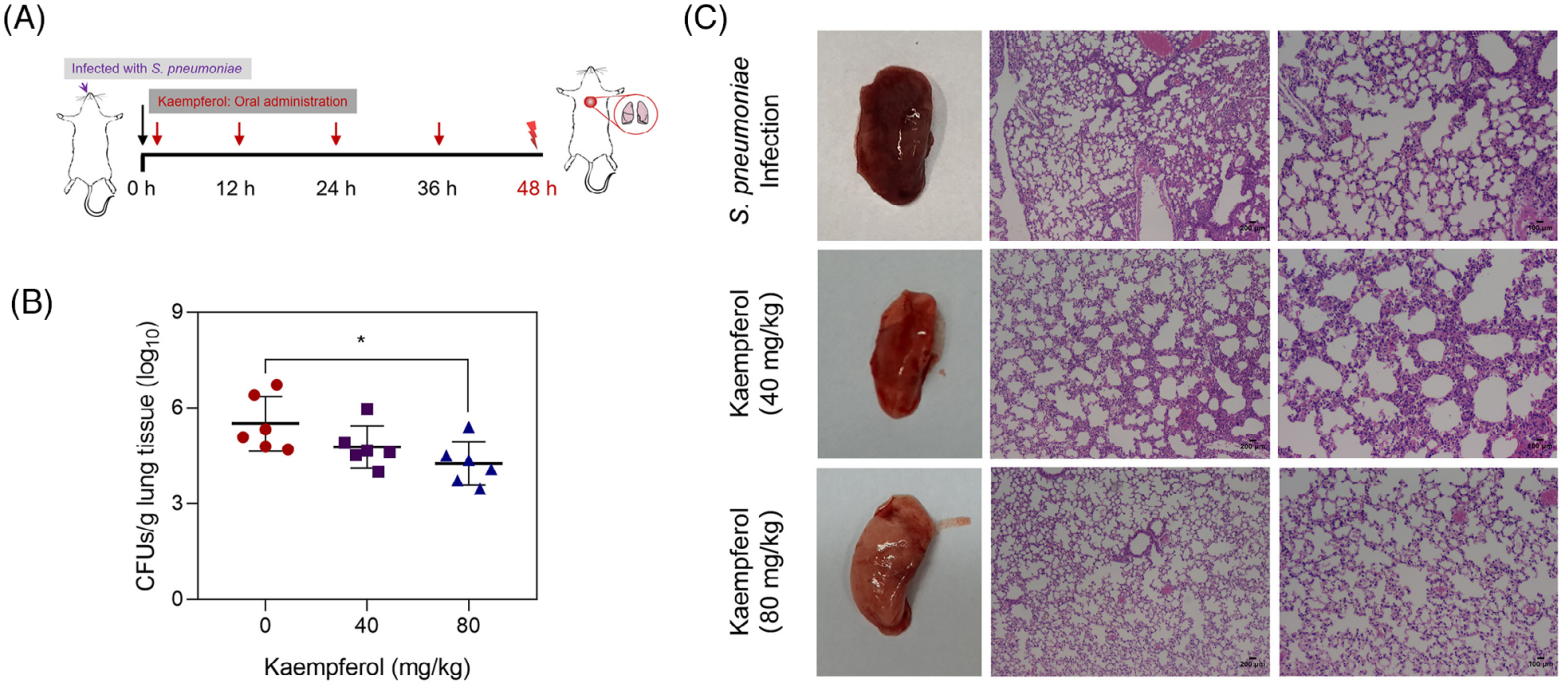
(A) Scheme of the experimental procedures of mice pulmonary infection by *Streptococcus pneumoniae*. (B) Bacterial burden in the lungs of mice (*n* = 6 per group) determined by microbiological plating. (C) Pathological observation of lungs in different groups were evaluated by H&E staining. Scale bar = 100 and 200 µm *p < 0.05. [Copyright:  Reproduced from [41] with kind permission from Institut Pasteur, 2022. Published by Elsevier Masson SAS.

Research stalled for a few years, but in 2023 following the COVID‐19 global health crisis scholars in China reported the discovery that kaempferol exerts powerful antibacterial action against *Streptococcus pneumoniae* by binding catalytic active sites of pneumolysin (PLY) as well as to those of sortase A (SrtA) peptidase [41]. After identifying the powerful anti‐biofilm activity in vitro inhibition activity due to inhibition of the aforementioned two key virulence factors, the team first reported how kaempferol oral administration to rats could significantly alleviate *S. pneumoniae* virulence in vivo (Figure 13).

Administering infected rats with kaempferol at 80 mg/kg dosage, resulted in significant decrease in the bacterial burden in lungs compared with the *S. pneumoniae*‐infected control (Figure 13B), with significant reduction of lung pathological injury (dark redness of macroscopic characteristic, hyperemia, edema and accumulation of inflammatory cells in lung tissues), in the lungs of rats treated with kaempferol (Figure 13C). The team concluded how kaempferol therapy shows “great potential for combating co‐infections and secondary bacterial infections.”

### 2.5 Quercetin and Rutin

**FIGURE 14:**
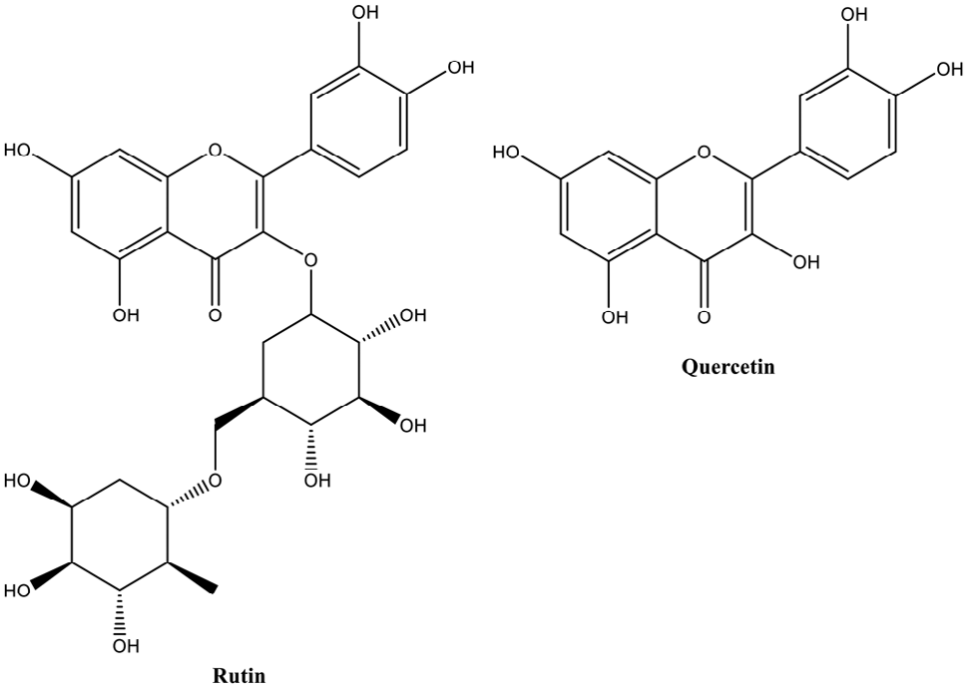
Chemical structures of rutin (left) and its aglycone quercetin (3,3,4,5,7‐pentahydroxiflavone).

Rutin (quercetin‐3‐rutinoside; Figure 14) is a flavanol found in numerous *Citrus* fruits, and in slightly higher amount in the peels of orange. For comparison, rutin is present in the concentration order of 3.3–3.9–4.7 mg/g of dried peel of lemon, clementine and orange fruits, respectively, whereas the most abundant flavonoid extractable from the peels of the same *Citrus* fruits is flavanone hesperidin present in 280–498–673 mg/g, in the peels of lemon, orange and clementine fruits, respectively [42].

Studying the low level of antibacterial activity of rutin against *E. coli*, which was restricted to permeable *E. coli* strains only, France's pharmaceutical industry researchers working in 1997 first showed that rutin (extracted from cottonseed flour) was highly active in stimulating topoisomerase IV‐dependent DNA cleavage (CC_50_ = 1 µg/mL) [43]. Topoisomerase IV is essential for cell survival and is along with gyrase one of the two targets for quinolones antibiotics. Five years later, scholars in Japan reported that although rutin did not show activity in itself, the antibacterial activity of kaempferol against *Salmonella enteritidis* and *Bacillus cereus* markedly decreased by the addition of rutin [44].

In detail, the MIC value for kaempferol against *S. enteritidis* went from 400 to 50 µg/mL and from 800 to 400 µg/mL in the case of *B. cereus*. The same team demonstrated that the combinations of quercetin and rutin was much more active than either flavonoid alone, with the MIC of quercetin against *S. enteritidis* going from 250 to 100 µg/mL and from 350 to 200 µg/mL in the case of *B. cereus*. Quercetin exerts moderate to good antibacterial activity against both Gram‐positive and Gram‐negative bacteria, though in general, it is more effective against Gram‐positive bacteria [45]. The mechanism of its antimicrobial action once again is multifactorial and includes cell membrane damage, change of membrane permeability, inhibition of synthesis of nucleic acids and proteins, reduction of expression of virulence factors, and preventing biofilm formation [45]. Reviewing its use as antimicrobial agent concluded that further work is required to enhance its bioavailability and unleash its therapeutic potential due the poor oral bioavailability and absorption of quercetin in the human body.

### 2.6 Eriodictyol and Eriocitrin

**FIGURE 15:**
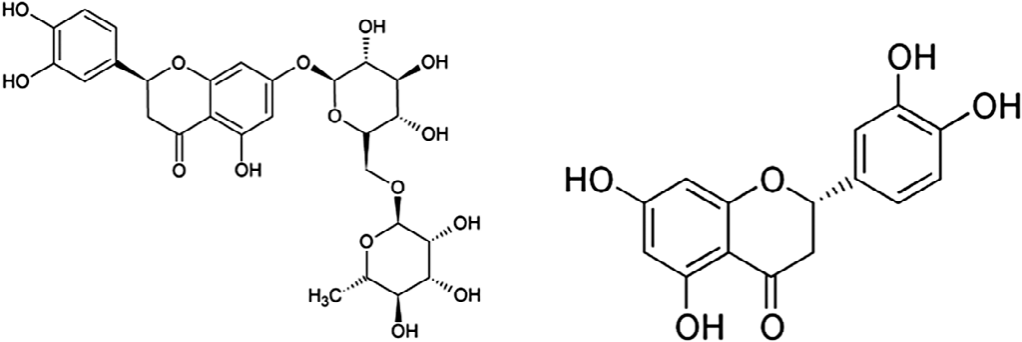
Chemical structures of eriocitrin (left) and eriodictyol.

Eriodictyol is the aglycone of eriocitrin (eriodictyol‐7‐*O*‐rutinoside) (Figure 15). Along with hesperidin, the latter flavanone is the most abundant flavonoid in lemon peel (84 mg of hesperidin and 176 mg of eriocitrin in 100 g of fresh weight peel) [46], being present also in relatively good amount in the bergamot peel.

Studying the antimicrobial effects of bergamot peel ethanolic extracts against Gram‐negative bacteria (*E. coli*, *Pseudomonas putida*, *Salmonella enterica*), Gram‐positive bacteria (*Listeria innocua*, *Bacillus subtilis*, *S. aureus*, *Lactococcus lactis*) and the yeast *Saccharomyces cerevisiae*, Mandalari et al. reported activity against all the Gram‐negative bacteria tested, and that their antimicrobial potency increased after enzymatic deglycosylation [7]. Eriodictyol showed the greatest activity amid all flavonoids tested (neohesperidin, hesperetin, neoeriocitrin, eriodictyol, naringin and naringenin) with MICs in the range of 250 and 800 µg/mL. Synergism was observed between eriodictyol and hesperetin against *E. coli* and *S. enterica*, and between eriodictyol and naringenin against *S. enterica* and *P. putida*. Synergism was ascribed by the team to the “combined reaction with the cell membrane as a possible primary target site but with different mode of inhibitory action.”

Surprisingly, these findings were not further explored nor has the antibacterial activity of eriocitrin investigated even though in 2023 researchers in Iran reported that molecular docking calculations clearly indicate the ability of eriocitrin to inhibit the OXA‐23 enzyme receptors of *A. baumannii* (an opportunistic pathogen that can cause a range of infections in humans, especially in hospitals) [47]. β‐Lactamase enzyme OXA‐23 encoded by OXA‐23 gene in *A. baumannii* belongs to the class D β‐lactamases and is responsible for conferring resistance to β‐lactam antibiotics, commonly used as a last resort for treating multidrug‐resistant bacterial infections. The presence of the OXA‐23 gene in *A. baumannii* strains allows them to produce the OXA‐23 enzyme, which can degrade and inactivate these antibiotics, leading to treatment failure and the spread of carbapenem‐resistant. The outcomes of the docking process of eriocitrin to 4K0X binding site of OXA‐23 enzyme performed using AutoDockVina software, show that eriocitrin is involved in hydrogen bonding with an affinity of −9.4 with the amino acids aspartic acid, glycine, threonine, alanine, serine, histidine, and glutamine.

### 2.7 Vicenin‐2 and Diosmin

**FIGURE 16:**
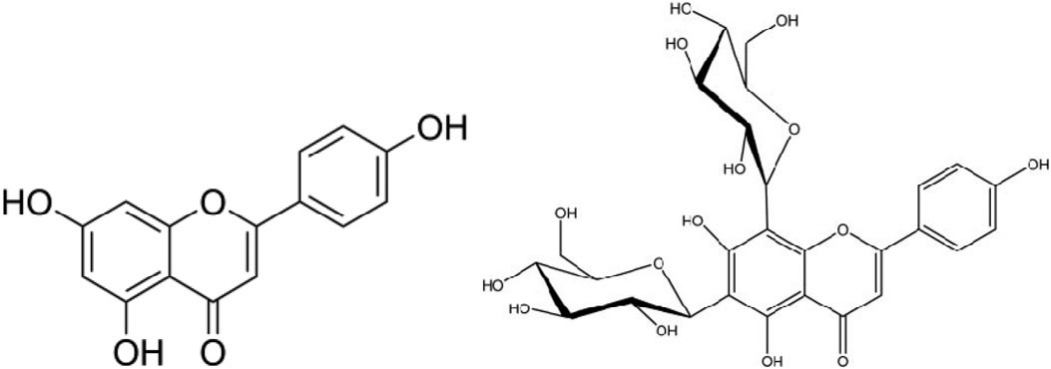
Chemical structures of apigenin (left) and apigenin‐7‐*O*‐glucoside (right).

Nearly all in glycosylated form and concentrated in the fruit (and thus in the juice), flavones are the second most abundant flavonoids in *Citrus* fruits after flavanones. In sweet orange juice from different cultivars, for example, apigenin‐6,8‐di‐C‐glucoside (vicenin‐2) is the most abundant flavone glycoside, with concentrations ranging from 25.8 to 69 mg/L [48]. The same apigenin (Figure 16) glycoside is also relatively abundant in other *Citrus* fruits, including lemon, grapefruit and tangerine.

**FIGURE 17:**
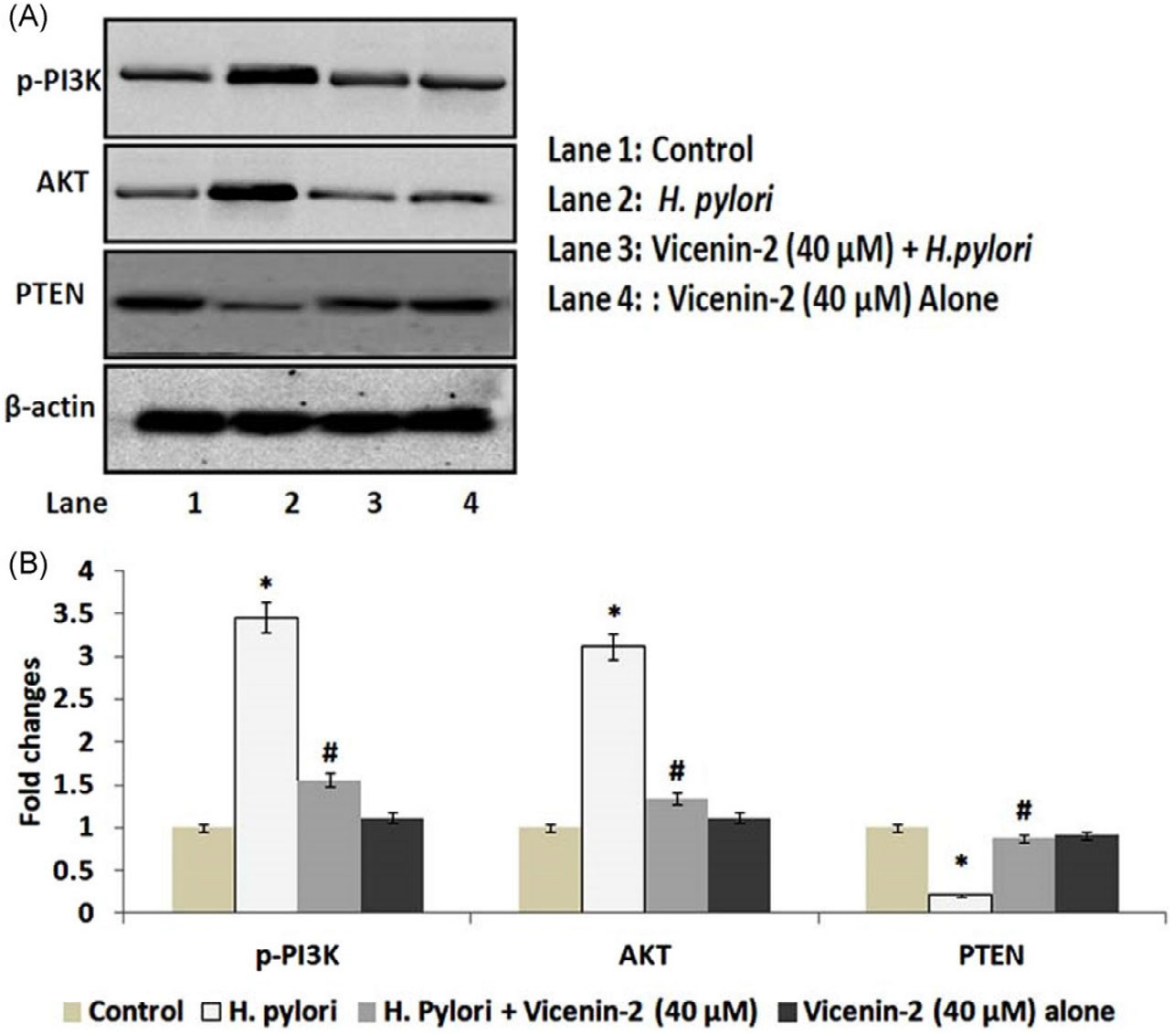
Vicenin‐2 on PI3K, AKT, and PTEN signaling in *Helicobacter pylori*‐infected GES‐1 cells. (A) Western blot analysis of PI3K, AKT, and PTEN expression in vicenin‐2 and/or *H. pylori*‐infected GES‐1 cells; β‐actin was used as a normal loading control. (B) The quantification of protein was performed by densitometric analysis using ImageJ software. The densitometry data represent means ± SD from three immunoblots and are shown as the comparative density of protein bands normalized to β‐actin. Values not sharing common marking differ significantly at ^*,#^ *p* ≤ 0.05 (Duncan's multiple range test). AKT, protein kinase B; PI3K, phosphatidylinositol 3‐kinase; PTEN, phosphatase and tensin homolog. Reproduced from [49], with kind permission.

Showing good inhibitory effect on *H. pylori* infection‐induced gastric carcinogenic signaling, the role of vicenin‐2 on *H. pylori* infection‐mediated gastric injury and gastric carcinogenic signaling events has recently been identified [49]. In brief, vicenin‐2 inhibits the *H. pylori* infection associated gastric carcinogenic events through modulation of phosphatidylinositol 3‐kinase (PI3K)/protein kinase B (AKT) and nuclear factor erythroid‐related factor‐2 (Nrf2) signaling in GES‐1 gastric cells. In closer detail, the range of protein expression for p‐PI3K, AKT, and PTEN levels demonstrated by quantitative western blot analysis shows evidence (Figure 17) that *H. pylori* co‐culture with GES‐1 cells resulted in increased protein expression ranges for the markers of p‐P13K, AKT, and decreased protein marker expression in the tumor suppressor tensin homolog PTEN. However, vicenin‐2 treatment displayed decreased protein expression ranges for the markers of p‐P13K, AKT, and consequently, PTEN expression was restored in *H. pylori*‐infected cells.

This seminal work identified vicenin‐2 as antibacterial agent specifically targeting *H. pylori* without causing damage to the normal gut flora as it happens with most antibiotics, resulting in severe gastrointestinal side effects. Therefore, the identification of drugs that could and decrease the incidence of infection is one of the vital concerns of the research society. As mentioned above, vicenin‐2 is particularly abundant in certain *Citrus sinensis* cultivars [48], and also in bergamot cultivars [50].

**FIGURE 18:**
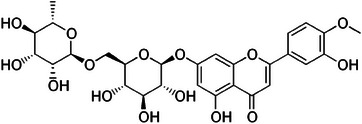
Chemical structures of diosmin.

Diosmin (3′,5,7‐trihydroxy‐4′‐methoxyflavone; Figure 18), a glucoside consisting of diosmetin substituted at position 7 by a rhamnose moiety, is another flavone relatively abundant in the flavedo and albedo (but not in the pulp) of *Citrus* fruits, particularly limes (*Citrus aurantifolia* and *Citrus limettioides*), citron (*Citrus medica*) and immature lemon (particularly the Meyer hybrid, up to 3 g/100 g dry weight) [51].

The glycoside shows antimicrobial activity, particularly against both Gram‐positive bacteria. In detail, diosmin had both inhibitory and bactericidal activity against six tested Gram‐positive bacteria, with the lowest MIC and MBC against *S. aureus* ATCC 25923 (in each case, 64 µg/mL). The activity against *S. aureus* ATCC 29213 and 43300, *S. epidermidis* ATCC 12228, *E. faecalis* ATCC 29212 and 51299 was in the 128–256 µg/mL range. No inhibitory activity was observed for any of the Gram‐negative bacteria tested [52].

**FIGURE 19:**
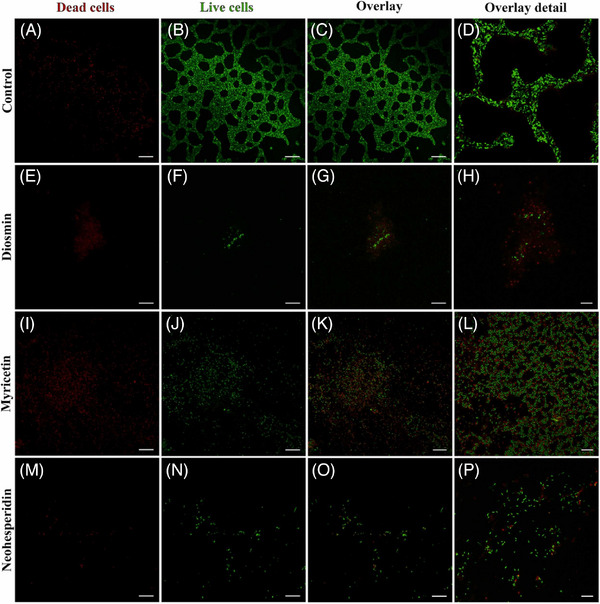
Representative CLSM images of *Pseudomonas aeruginosa* PAO1 biofilms in control conditions (A–D) and after treatment with diosmin (E–H), myricetin (I–L), and neohesperidin (M–P). Bar = 25 µm for A, B, C, E, F, G, I, J, K, M, N, and O; bar = 10 µm for D, H, L, and P. Reproduced from [53], Creative Commons Attribution (CC BY) license from The Authors, 2024.

On the other hand, scholars in Serbia recently reported that diosmin is an antibacterial agent that effectively inhibits *P. aeruginosa* infections with MIC and MBC values toward *P. aeruginosa* ATCC 27853 (the most resistant strain to the treatment) of 0.4 and 0.8 mg/mL [53]. For comparison, streptomycin has MIC 0.05 mg/mL and MBC 0.1 mg/mL. Biofilm treatment with diosmin resulted in the lowest percentage of live microbial cells observed by confocal laser scanning microscopy (CLSM) live/dead cell imaging, including significant reduction in biofilm biomass, exopolysaccharide and eDNA production. A significantly lower number of cells (both live and dead) can be detected, especially in the presence of diosmin and neohesperidin as well as the absence of a regular biofilm structure that can be noticed in the control sample (Figure 19).

**FIGURE 20:**
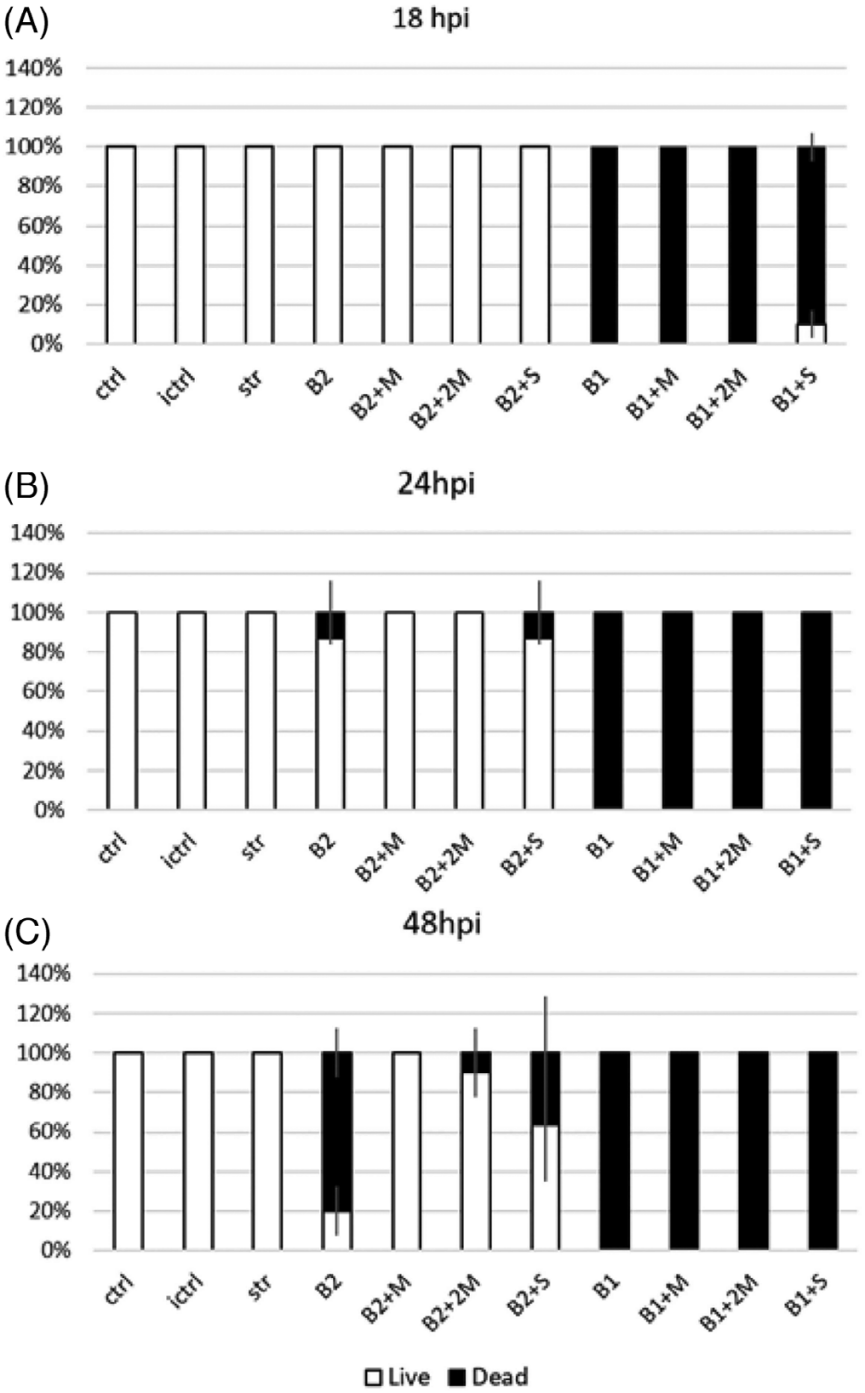
Protective effect of diosmin on survival of embryos after 18, 24, and 48 h post infection (hpi, A, B and C, respectively) with *Pseudomonas aeruginosa* PAO1 (mean ± SE): ctrl—control group; ictrl—control group with injured tail; B1—10^7^ CFU/mL; B2—10^6^ CFU/mL; M—MIC (0.1 mg/mL); 2M—2 MIC (0.2 mg/mL); str—streptomycin (10 µg/mL). Reproduced from [53], Creative Commons Attribution (CC BY) license from The Authors, 2024.

The team, furthermore, found via in vivo toxicity tests of the flavonoids studied in *P. aeruginosa* zebrafish embryo infection model, that diosmin stood out as non‐embryotoxic. Whereas exposure of embryos to high inoculum of 10^7^ CFU/mL resulted in 100 % mortality of exposed embryos regardless of the applied treatment (both tested concentrations of diosmin, MIC and MBC, or antibiotic control streptomycin), exposure to lower inoculum (10^6^ CFU/mL) in the presence of diosmin in concentrations equivalent to MIC (0.1 mg/mL) and MBC (0.2 mg/mL) resulted in significantly improved survival of injured embryos in comparison with untreated group (Figure 20).

Ivanov et al. concluded that even though the antibacterial activity of diosmin is lower than that of streptomycin, diosmin could be used in combination with commercial antibiotics to enhance antimicrobial potency at lower and safer concentrations of commercial antibiotics, the majority of which are associated with different adverse effects [54].

## 3 Outlook and Perspective

Relatively recent research started in the early 2000s has clearly unveiled the antimicrobial activity of citrus flavonoids: naringenin, hesperidin, apigenin, diosmin, rutin, vicenin‐2, quercetin, nobiletin, tangeretin, eriodictyol are all powerful antibacterial and often also antifungal species. Two *Citrus* flavonoid‐based antimicrobials, a moutwash and surface sanitizer based on flavonoids, were commercialized in the early 2010s. Both are water‐soluble formulations containing nine flavonoids extracted from *Citrus aurantium* in which flavonoids are combined with organic acids, possessing strong antimicrobial, anti‐inflammatory, and antioxidative properties [5].

Citrus flavonoids such as rutin, quercetin, hesperidin and diosmin have been extensively tested in clinical trials against Type II diabetes, with relevant results that suggest employment of rutin and hesperidin and diosmin in management on the illness [55]. A recent review and dose–response meta‐analysis from eight randomized clinical trials (RCTs) concerning the impact of citrus flavonoids supplementation (CFS) on endothelial function clearly indicates that CFS enhances endothelial function [56]. A recent RCT found that an orange peel extract rich in lipophilic flavonoids (PMFs) provides better results in terms of hemostasis, pain relief, and palatal wound healing compared to Alveogyl state‐of‐the art commercial wound dressing material (orange peel is extracted with dichloromethane; the extract is dried and mixed with cyclodextrin maintaining a 3:1 ratio of β‐cyclodextrin to orange peel extract as a wound dressing material) [57].

To the best of our knowledge sections, no clinical trial on the antimicrobial properties of flavonoids, and *Citrus* flavonoids in particular, has been reported in the scientific literature, confirming what Song et al. noted in 2021: “flavonoids are underappreciated antibacterials with large potential to circumvent the existing antibiotic resistance” [9]. One of the reasons explaining said under appreciation of *Citrus* flavonoids as antibacterials is the extremely low water solubility of many *Citrus* flavonoids that affects their biological activity in vivo [58].

Looking at forthcoming clinical trials, thus, it is instructive to learn that to overcome said extremely low water solubility, eriocitrin at 70wt%, along with 5% hesperidin, 4% naringin, 1% didymin, and 20% of plant fiber material, chiefly consisting of lignin, pectin and cellulose was commercialized in the mid 2010s for managing hyperglycemia and reversal of prediabetes condition [59]. A double‐blind, randomized, placebo‐controlled trial conducted in Brazil over 12 weeks between late 2017 and early 2018 on 120 individuals (30 given a daily dose of 400 mg placebo; 30 a daily dose of 200 mg Eriomin; 30 given a daily dose of 400 mg Eriomin; 30 subjects given a daily dose of 800 mg Eriomin) 103 of which previously classified as prediabetic, gave remarkable results with 27% of subjects supplemented with Eriomin 200 having reversed prediabetes to the normal condition (euglycemia). The dispersion of eriocitrin over the fiber material is key to enhance its bioavailability making the same nutraceutical product effective also reducing the growth of microorganisms associated with intestinal dysbiosis and increased the abundance of beneficial bacteria (eventually affording a lower glycemic level and a 22% increased production of glucagon‐like peptide‐1 along with significant beneficial effects on the microbiota in subjects with prediabetes) [60].

**FIGURE 21:**
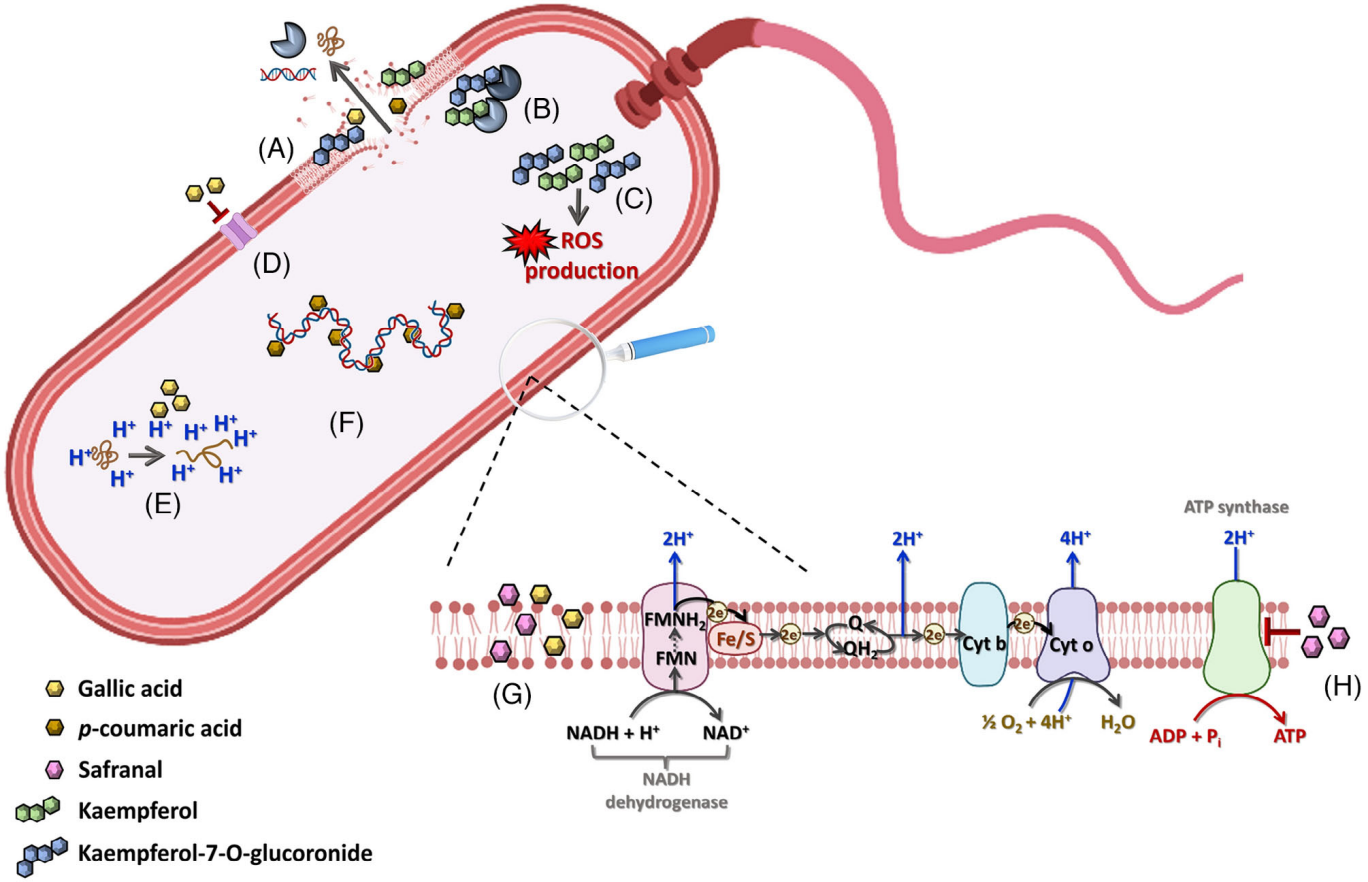
Mechanism of action of lemon IntegroPectin films against Gram‐negative bacterial strains: (A) disruption of membrane with consequent leakage of macromolecules in the extracellular environment, (B) targeting of enzymes crucial for cell functioning and vitality, (C) ROS production, (D) inhibition of efflux pumps, (E) acidification of the cytoplasm and protein denaturation, (F) intercalation with the DNA double helix, (G) permeabilization of the bacterial membrane, and (H) inhibition of ATP synthase. Reproduced from [62], Creative Commons Attribution (CC BY) license, from The Authors, 2022.

Said enhanced delivery partly explains the exceptional broad‐scope antibacterial activity of grapefruit and lemon IntegroPectin [61]. These newly extracted pectic bioconjugates sourced via cavitation from citrus processing waste dispersed in water, indeed, carry flavonoids such as naringin, hesperidin and kaempferol that are delivered via sustained release of flavonoids within the bacterial cells [62]. Compared to commercial *Citrus* pectin sourced with mineral acid having high degree of methylation (DM), containing no flavonoids or terpenes and nearly free of native RG‐I rhamnogalacturonan regions, the newly isolated IntegroPectin has very low DM, is rich in RG‐I lateral chains as well as of terpenes and flavonoids. Combined with the intrinsic antimicrobial activity of pectin (particularly, against Gram‐negative bacteria, yeasts and nonfilamentous fungi) [63], the unique structure of these new bioconjugates ensures a combined mechanism of action that explains the broad scope bactericidal activity of IntegroPectin against both Gram‐positive and Gram‐negative bacteria (Figure 21).

Though varying amid different flavonoids, the mechanisms of action of *Citrus* flavonoids seem to include multiple modes of action including inhibition of exopolysaccharides, quorum sensing, generation of ROS at the bacterial surface, cell membrane damage, change of membrane permeability, inhibition of synthesis of nucleic acids and proteins, reduction of expression of virulence factors, and mitochondrial dysfunction.

Another key finding of the researches on the antimicrobial properties of *Citrus* flavonoids is that most often they can show synergism, such as first in the case of eriodictyol when synergism was observed between eriodictyol and hesperetin against *E. coli* and *S. enterica*, and between eriodictyol and naringenin against *S. enterica* and *P. putida*, likely due to the combined reaction with the cell membrane, but with different mode of inhibitory action [7]. This is of the uttermost importance because thanks to today's advanced chemical separation techniques pure flavonoids can be readily obtained, mixing flavonoids that have high activity against Gram‐positive bacteria such as naringenin, hesperedin, or quercetin with others showing good activity again Gram‐negative bacteria such as eriodictyol, kaempferol, apigenin, and diosmin. The same approach was actually followed developing the commercial “Citrox” soluble formulation containing nine flavonoids extracted from *Citrus aurantium* to further enhance the antimicrobial properties of the single flavonoids [6].

In conclusion, the broad‐scope antimicrobial activity of many *Citrus* flavonoids, along with the fact that they exert their antimicrobial activity through multifactorial mechanisms alternative to microbial enzyme inhibition of most commercial antibiotics, is highly promising toward the development of new generation antimicrobials for human and animal healthcare without driving multidrug resistance. The long‐researched issue of their poor bioavailability due to low or lack of solubility in water has been lately resolved by heterogenizing them on carbohydrate fibers, on β‐cyclodextrin or directly on new sourced *Citrus* pectin, to which flavonoids actually bind during the cavitation‐based extraction [64]. Forthcoming RCTs will be eased by the fact that many *Citrus* flavonoids are safe for human and animal consumption, with many holding the generally recognized as safe (GRAS) status that enables their widespread use in nutraceutical and food products.

## Conflicts of Interest

The authors declare no conflicts of interest.

## Data Availability

The authors have nothing to report.
